# Viral Restriction Activity of Feline BST2 Is Independent of Its N-Glycosylation and Induction of NF-κB Activation

**DOI:** 10.1371/journal.pone.0138190

**Published:** 2015-09-17

**Authors:** Weiran Wang, Jiawen Wang, Meng Qu, Xiaojun Li, Jingyao Zhang, Haihong Zhang, Jiaxin Wu, Bin Yu, Hui Wu, Wei Kong, Xianghui Yu

**Affiliations:** 1 National Engineering Laboratory for AIDS Vaccine, School of Life Science, Jilin University, Changchun, Jilin Province, People’s Republic of China; 2 Key Laboratory for Molecular Enzymology and Engineering of the Ministry of Education, School of Life Science, Jilin University, Changchun, Jilin Province, People’s Republic of China; 3 Center for New Medicine Research, Changchun University of Chinese Medicine, Changchun, Jilin Province, People’s Republic of China; Helmholtz Zentrum München - German Research Center for Environmental Health, GERMANY

## Abstract

BST2 (CD317, tetherin, HM1.24) is an interferon-inducible transmembrane protein which can directly inhibit the release of enveloped virus particles from infected cells, and its anti-viral activity is reported to be related to the specific topological arrangement of its four structural domains. The N-terminal cytoplasmic tail of feline BST2 (fBST2) is characterized by a shorter N-terminal region compared to those of other known homologs. In this study, we investigated the functional impact of modifying the cytoplasmic tail region of fBST2 and its molecular mechanism. The fBST2 protein with the addition of a peptide at the N-terminus retained anti-release activity against human immunodeficiency virus type-1 and pseudovirus based on feline immunodeficiency virus at a weaker level compared with the wild-type fBST2. However, the fBST2 protein with addition of a peptide internally in the ectodomain proximal to the GPI anchor still retained its anti-viral activity well. Notably, the N-glycosylation state and the cell surface level of the N-terminally modified variants were unlike those of the wild-type protein, while no difference was observed in their intracellular localizations. However, in contrast to human BST2, the wild-type fBST2 did not show the ability to activate NF-κB. Consistent with previous reports, our findings showed that adding a peptide in the cytoplasmic tail region of fBST2 may influence its anti-viral activity. The shorter N-terminal cytoplasmic region of fBST2 compared with human BST2 did not apparently affect its anti-viral activity, which is independent of its N-glycosylation and ability to activate NF-κB.

## Introduction

Lentiviruses, which belong to a subfamily of retroviruses, can infect T cells and cause slow disease progression. The domestic cat lineage has faced invasions by retroviruses such as feline immunodeficiency virus (FIV), feline leukemia virus (FeLV) and feline foamy virus (FFV). Cats also harbor the endogenous RD114 gamma retrovirus [1, 2] and full-length endogenous FeLVs [3]. FIV shares several relevant features [4] with the human immunodeficiency virus (HIV). Unlike the simian immunodeficiency virus infecting African green monkeys (SIVagm) and other naturally occurring SIVs in their natural hosts [5], FIV can lead to a high level of immune stimulation in *Felis catus* [6, 7] and an immunodeficiency syndrome similar to that caused by HIV-1 in humans. In addition, FIV enters T cells via CD134 [8] and CXCR4 [9–11], and like HIV-1 its genome encodes the Vif protein, which is necessary for the production of fully infectious virus [12]. HIV and FIV have similarities in genome structure, mechanism of transmission, course of infection and pathogenicity. Therefore, the domestic cat is considered the smallest available natural animal model for the study of acquired immunodeficiency syndrome (AIDS) in humans and for the development of potential therapeutic strategies [10, 11, 13].

Retroviruses exploit numerous positively acting cellular factors and pathways to maximize viral particle production [14]. However, in addition to conventional innate and acquired immune responses, humans and other mammals also have multiple systems to suppress virus replication through the actions of innate host cell restriction factors [15]. These host cellular proteins are constitutively expressed or induced by interferon (IFN) in response to viral infection. Host restriction factors represent a crucial aspect of innate immunity, defined as intrinsic immunity [16, 17]. IFN-inducible factors restricting viral replication include the cytidine deaminase APOBEC3 family proteins [18–20] and the E3 ubiquitin ligase TRIM5 [21–23], which target replication primarily during the process of viral entry. The third IFN-inducible factor, BST2 (also known as tetherin, CD317 and HM1.24), acts to restrict viral release [24–28]. The recently identified SAMHD1 protein, which is also considered a restriction factor, suppresses the viral genome replication process [29–32]. Viruses in turn have evolved to express adaptor molecules that antagonize these host cell restrictions, thereby allowing their replication to proceed efficiently. For example, lentiviral Vif proteins [33, 34] and spumaviral Bet proteins [35–37] counteract APOBEC3s; meanwhile, HIV-1 Vpu, SIV Nef, and HIV-2 and SIV Envs may counteract BST2s [24–26, 38–41], and the Vpx protein induces proteasomal degradation of SAMHD1 [42, 43].

Human BST2 is a 28- to 36-kDa type II single-pass transmembrane (TM) protein. It is anchored to the cell membrane by both an N-terminal transmembrane domain and C-terminal glycophosphatidylinositol (GPI) anchor or other type of C-terminal membrane anchor such as C-terminal hydrophobic residue tryptophan, which are linked by an extracellular coiled-coil domain that promotes dimerization of adjacent BST2 molecules [44]. Accordingly, BST2 in both the cell membrane and the envelope of the budding virus can prevent virus release either by direct cross-linking or by the formation of dimers between adjacent coiled-coil domains. Two potential N-linked glycosylation sites and three conserved cysteine residues are present in the extracellular domain. An artificial BST2-like protein, assembled in a similar configuration as the transferrin receptor (TfR), dystrophia myotonica protein kinase (DMPK) and urokinase Plasminogen Activator Receptor (uPAR), is able to mimic the biological activity of the native human BST2 [28]. Additionally, human BST2 was identified as an inducer of nuclear factor-kappa B (NF-κB) activation in a whole-genome transfection screen [45]. The critical motif for its NF-κB signaling activity, which is mainly located in the cytoplasmic and ectodomain, includes Y6, Y8, RVP (10–12), three cysteines (53, 63, 91), two asparagines (65, 92), L70 and L123 [46–48]. Moreover, the deletion of the GPI anchor can also reduce this cellular function [46]. Human BST2 exhibits broad anti-viral activity against a wide range of enveloped viruses, such as HIV-1, HIV-2, SIV and other retroviruses, as well as Lassa, Marburg and Ebola virus-like particles [49–51]. During the late phase of the viral replication pathway, BST2 causes nascent viruses to remain trapped at the surface of the infected cell and to accumulate thereafter in endosomes following internalization [24–26]. Some reports have claimed several distinct functions of the cytoplasmic tail domain of human BST2. The deletion of each structural domain on the N-terminal HA-tagged human BST2 was initially reported to result in severe impairment of its anti-HIV-1 virus release function [24]. However, recent studies indicated that fBST2 has a very short N-terminal cytoplasmic region compared with those of other mammalian and non-mammalian homologs. Surprisingly, it was shown to significantly block budding of FIV, HIV-1, HIV-2 ROD10, SIVmac239 and RD-114 particles [52, 53]. An artificial fBST2 mutant containing a longer N-terminal cytoplasmic homolog only exhibited limited anti-viral capability [52, 54]. Moreover, ovine BST2A, one of the two ovine BST2 isoforms, also retains a cytoplasmic tail shorter than that of human BST2. Interestingly, this shorter isoform was found to display a stronger anti-viral activity than the longer isoform [55]. Cocka and Bates showed that naturally occurring variants of human BST2 initiating from the internal ATG were partially resistant to Vpu [47]. Another recent study showed that the cytoplasmic tail of human BST2 is unnecessary for restricting HIV-1 but important for inducing intracellular signaling, and an N-terminal or internal HA-tagged BST2 was found to cause the loss of functions of anti-virus release and NF-κB activation [56]. Based on these observations, we hypothesized that the shorter N-terminal cytoplasmic region of the fBST2 protein may lack the ability to induce NF-κB activation, and the HA tag can also compromise the functions of fBST2.

In this study, we explored the impact of HA-tagged fBST2 and its longer N-terminal cytoplasmic homolog on its anti-viral function against HIV-1 and FIV pseudovirus. The fBST2 protein with a shorter cytoplasmic tail (wild-type) exhibited greater anti-viral activity against HIV-1 and FIV-ΔVif-ΔOrfA-ΔEnv-GFP than did the variants. In addition, fusion of the HA peptide to the N-terminus of fBST2 or internally in the ectodomain impaired its anti-viral activity to varying degrees. Notably, its cell surface expression and N-glycosylation state changed dramatically. These observations suggest that the shorter cytoplasmic tail of fBST2 accounts for its stronger anti-release activity. Additionally, the wild-type fBST2 does not have the function of inducing NF-κB activation, which is not directly related to the anti-release activity. These results provide further insight into the function of human BST2.

## Materials and Methods

### Cell cultures and transfections

HEK293T (ATCC, no. CRL-11268) and CrFK (ATCC, no. CCL-94) cells were cultured and maintained in Dulbecco’s modified Eagle’s medium (DMEM) supplemented with 10% fetal bovine serum (FBS) at 37°C and 5% CO_2_. Transient plasmid transfections into 293T cells and CrFK cells were performed using Lipofectamine 2000 (Invitrogen, Carlsbad, CA, USA) according to the manufacturer’s instructions.

### Plasmids

Total RNA was extracted from feline CrFK cells using TRIzol (Invitrogen) separately. The fBST2* and fBST2 sequences designed as previously described [52] were obtained by RT-PCR. All of the fBST2 variants, including fBST2-IHA, fBST2-NHA, fBST2 N79A, fBST2 N119A, fBST2 N79/119A and fBST2 ectodomain, were engineered based on fBST2 by using the QuickChange mutagenesis system (Agilent, Santa Clara, CA, USA). The eukaryotic expression plasmids expressing fBST2*, fBST2, fBST2-IHA, fBST2-NHA, fBST2 N79A, fBST2 N119A and fBST2 N79/119A were cloned into the VR1012 vector, and the prokaryotic expression plasmid expressing the fBST2 ectodomain was cloned into the pET-26b (+) vector. All plasmids were confirmed by sequencing. All primers were synthesized by the solid phase phosphoramidite triester method (ShineGene, Shanghai, China). The HIV-1 proviral clone pNL4-3, FIV pseudovirus clone (pFP93 and pGINSIN), pEGFP-N3 and VR1012 were described previously [32, 57, 58].

### Antibodies

The following antibodies were used in this study: anti-HA mouse monoclonal antibody (mAb) (Covance, Emeryville, CA, USA), anti-tubulin mouse mAb (Covance), anti-His mouse mAb (Sigma, St. Louis, MO, USA) and anti-p24 mouse mAb obtained from an HIV-1 p24 hybridoma from the National Institutes of Health AIDS Research and Reference Reagent Program (NIH-ARRRP).

Alkaline phosphatase conjugated goat anti-rabbit or anti-mouse immunoglobulin G secondary antibodies were purchased from Jackson ImmunoResearch Laboratories (West Grove, PA, USA). Alexa Fluor 488 goat anti-mouse IgG secondary antibodies and Alexa Fluor 633 goat anti-rabbit IgG secondary antibodies were from Invitrogen.

### Protein purification and preparation of polyclonal antibody (pAb)

The fBST2 ectodomain-pET-26b (+) plasmid was transformed into *Escherichia coli* BL21, and the expressed proteins were found to be water soluble after induction with isopropyl-β-d-thiogalactoside (IPTG). Approximately 5 h after IPTG induction, cells were harvested in PBS and centrifuged at 6000 × *g* for 20 min and then resuspended with 50 mM Tris—HCl (pH 8.0), followed by ultrasonication for 20 min. The cell lysates were centrifuged at 10,000 × *g* for 30 min, and the supernatants were passed through a 0.45-μm filter before purification by immobilized metal ion affinity chromatography (IMAC). Each lysed sample was loaded onto a column containing the resin pre-equilibrated with washing buffer, and the column was washed with 10 bed volumes of wash buffer (150 mM Nacl, 50 mM Tris—HCl, pH 8.0) and increasing concentrations of imidazole (10, 20, 35 and 50 mM). The fBST2 ectodomain protein was eluted with 10 bed volumes of wash buffer containing 500 mM imidazole and concentrated with a 2.5-kDa ultrafiltration tube (Millipore, Billerica, MA, USA) to the final concentration of approximately 1 mg/ml.

For the first immunization (day 0), 0.4 mg of protein was emulsified with a same volume of Freund's complete adjuvant and injected into the back of the rabbit by subcutaneous injection. For the next three immunizations (days 14, 21 and 28), 0.4 mg of protein was blended with a same volume of Freund's incomplete adjuvant and injected into the back of the rabbit by subcutaneous injection. On day 29, serum of blood obtained from rabbit auricular veins was used for antibody detection by Western blot. A week after the final immunization (day 36), additional serum as anti-fBST2 pAb was separated from cardiac blood by centrifugation and frozen at -20°C.

### Western blotting

Cells were harvested at 48 h after transfection, washed once with pre-chilled phosphate-buffered saline (PBS) and lysed in RIPA buffer (150 mM NaCl, 50 mM Tris, 1% Triton X-100, 0.1% SDS) for 20 min at 4°C, followed by addition of 4× SDS sample buffer (1 M Tris, pH 6.8, with 8% SDS, 40% glycerol, 0.4 M dithiothreitol, and 0.8% bromophenol blue). The samples were subjected to SDS-PAGE after boiling for 10 min, and the proteins were transferred onto nitrocellulose membranes by semi-dry transfer (Bio-Rad, Hercules, CA, USA). After blocking in 5% non-fat milk for 10 min, the membranes were probed with various primary antibodies against proteins of interest overnight at 4°C. After incubation with secondary antibodies, immunoreactions were visualized with 5-bromo-4-chloro-3indolyl phosphate (BCIP) and nitro blue tetrazolium (NBT) solutions.

### Immunofluorescence analysis

HEK293T cells (40–60% confluent) grown on coverslips in a 24-well plate were transfected with plasmid DNA using Lipofectamine 2000. At 48 h after transfection, cells were fixed with 4% paraformaldehyde in PBS for 10 min at room temperature. For intracellular analysis, cells were permeabilized with 0.1% Triton X-100 in PBS for 8 min. Thereafter, the cells were washed three times with PBS, blocked with 10% FBS in PBS and then incubated with anti-fBST2 pAb diluted 1:500 or anti-HA mAb diluted 1:1000 in PBS containing 1% FBS for 1.5 h at room temperature. Cells were washed three times with PBS and stained with Alexa Fluor 633 goat anti-rabbit IgG (Invitrogen) or Alexa Fluor 488 goat anti-mouse IgG (Invitrogen) diluted 1:1000 in PBS (1% FBS) along with 1 mg/ml 4,6-diamidino-2-phenylindole (DAPI) for 40 min at room temperature. The cells were then washed three times in PBS and incubated with ER-Tracker (http://www.biomart.cn/genecopoeia/index.htm GeneCopoeia, Rockville, MD, USA) for 10 min at room temperature. For surface protein analysis, the same protocol was followed, except the Triton X-100 step was omitted. Stained cells were finally washed three times with PBS and subjected to detection. Fluorescent images were obtained using a Nikon ECLIPSE Ti system with a 20× objective.

### Virus production and infectivity assay

HIV-1 particles were produced by transient transfection of 293T cells in a 6-well plate with a proviral construct and indicated amounts of other plasmids. Forty-eight hours later, supernatants (2 ml per culture) from producer cells were harvested, clarified by centrifugation and passed through a 0.22-μm filter. The viral particles were concentrated through a 20% sucrose layer at 110,000 × *g* for 1.5 h and resuspended in 30 μl of RIPA buffer. Virus particle pellets and corresponding cell lysates were analyzed by SDS—PAGE and Western blot. In single-cycle infectivity assays, 50 ml of the filtered supernatant was mixed with DEAE-dextran (Sigma) at a final concentration of 15 mg/ml and incubated with TZM-bl indicator cells in a 96-well plate. At 48 h post-infection, the cells were lysed, mixed with luciferase substrates and assayed for luciferase activity using a fluorescence microplate reader to represent released virion yield.

The FIV-ΔVif-ΔOrfA-ΔEnv-GFP reporter virus was produced by transfecting CrFK cells with pFP93, pGINSIN and pVSV-G at a mass ratio of 3:3:1. Forty-eight hours later, supernatants (2 ml per culture) from producer cells were harvested, clarified by centrifugation and passed through a 0.22-μm filter. The viral particles were assayed for fluorescent intensity (GFP) using a fluorescence microplate reader to evaluate released virus yield. The cells also were harvested, and the fluorescent intensity was analyzed by flow cytometry to assess virus packaging.

### Peptide-N-Glycosidase F (PNGase F) digestion of fBST2

293T cells were transfected with wild-type or fBST2 mutants, lysed with 1% NP40 in PBS on ice for 5 min and then centrifuged to remove nuclei. Lysates were incubated in glycoprotein denaturing buffer at 100°C for 15 min to denature the proteins. The reaction was carried out in a total volume of 20 μl, including 2 μl 10× G7 reaction buffer, 2 μl 10% NP40 and 16 μl lysate, and incubated with or without 1000 U of PNGase F (New England Biolabs, Ipswich, MA, USA) at 37°C for 2 h prior to Western blot analysis with anti-fBST2 pAb.

### Flow cytometry analysis

293T and CrFK cells maintained in 6-well plates were transfected with plasmids along with the pEGFP-N3 vector (Clontech, Mountain View, CA, USA), which expresses EGFP as a marker of successful transfection, using Lipofectamine 2000. At 48 h after transfection, cells were fixed with 4% paraformaldehyde in PBS for 30 min at room temperature. The cells were then blocked with 10% FBS in PBS for 10 min and stained with an anti-fBST2 pAb, followed by Alexa Fluor 633 conjugated goat anti-rabbit IgG (Invitrogen). The stained cells were analyzed on a MoFlo XDP cell sorter (Beckman Coulter, Brea, CA, USA).

### Luciferase assay for detection of NF-κB activity

293T and CrFK cells maintained in 12-well plates were transfected with indicated BST2 expression plasmids along with the pNF-κB-Luc reporter plasmid (Agilent, Santa Clara, CA, USA). Forty-eight hours later, cells were disrupted in Triton X-100 lysis buffer. Lysates were transferred to a black flat bottom 96-well plate, and the Luciferase Assay System substrate (Promega Corporation, Madison, WI, USA) was added to the lysates according to the manufacturer's instructions. Samples were analyzed in a Luminoskan Ascent microplate luminometer (Thermo Scientific, Waltham, MA, USA).

### Statistical analysis

Group comparisons were performed using t-tests (and nonparametric tests). Calculations were performed with the INSTAT2 software package (GraphPad Software, San Diego, CA, USA). Results are given as means ± SEM. Statistical significance was accepted for *P* < 0.05.

## Results

### Construction of fBST2 variant expression vectors

**Fig 1:**
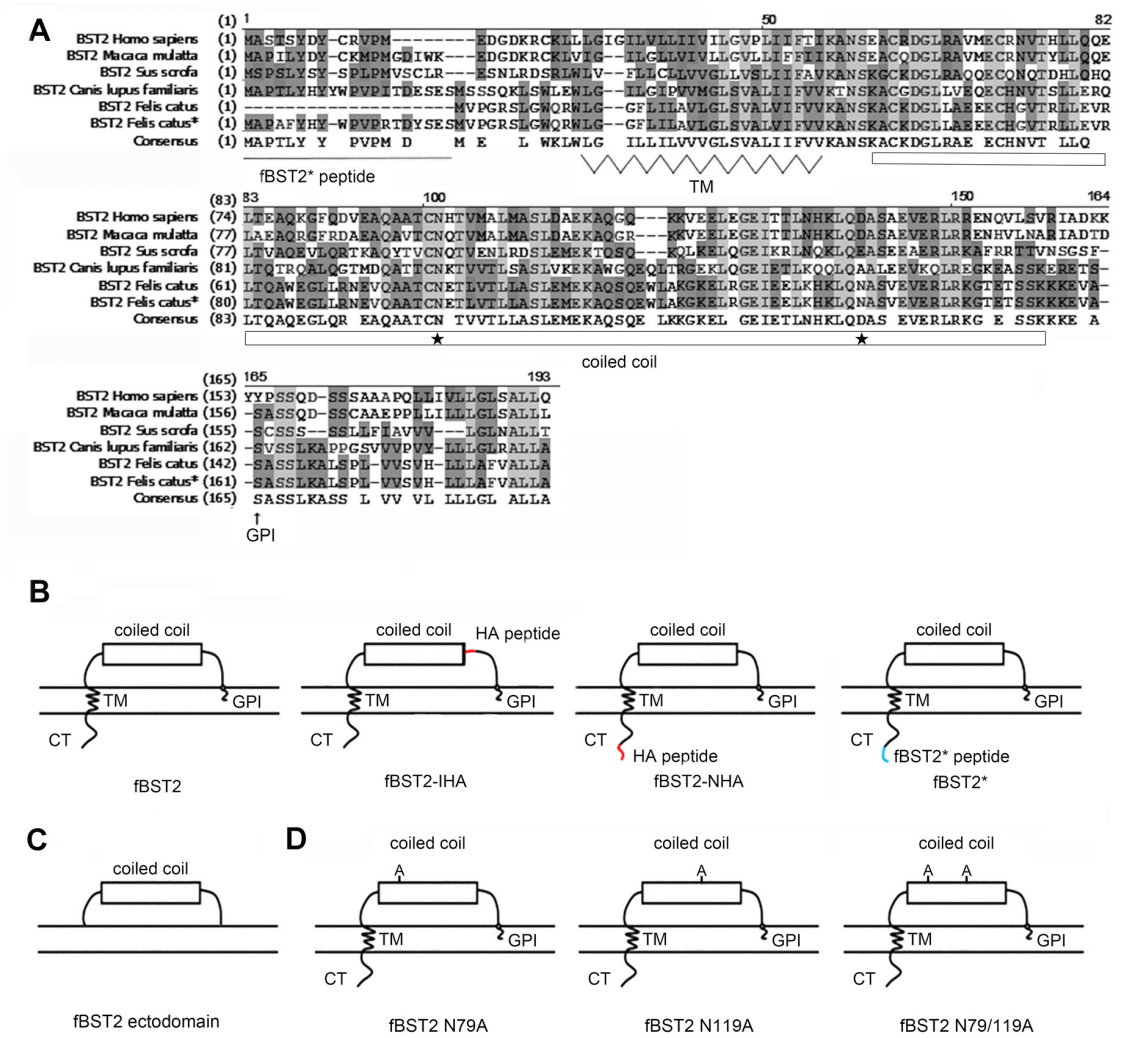
Amino acid sequence alignment and schematic representation of BST2s. (A) Amino acid sequence alignment of BST2s of H. sapiens, M. mulatta, S. scrofa, Canis lupus familiaris, F. catus and F. catus*. Identical amino acids are shaded in light grey, and conserved or similar residues are shown in dark gray. Dashes represent gaps. The transmembrane region, extracellular coiled-coil domain and GPI anchor, marked under the consensus sequences. The N-glycosylation sites in the extracellular coiled-coil domain are indicated by black stars. (B, C and D) Schematic representation of the fBST2 constructs used in this study.

As previously demonstrated, the fBST2 amino acid sequence showed a shorter N-terminal cytoplasmic tail compared with other BST2 from *Homo sapiens*, *Macaca mulatta* and *Sus scrofa* in an alignment (Fig 1A). However, fBST2 contains a 57 in-frame nucleotide sequence upstream of the first ATG, starting with a GAG codon that can potentially encode 19 amino acids (fBST2* peptide in Fig 1A), which displayed significant homology with the N-terminal regions of all the other BST2s analyzed. Indeed, fBST2 has been characterized with a short N-terminal region and shown to be counteracted by the FIV envelope glycoprotein [52, 54].

In order to investigate further the potential relevance of the fBST2 N-terminal region, we studied the impact of its lengthened homolog along the sequence upstream of the annotated methionine on the anti-viral function and other characteristics of this protein. First, we constructed an fBST2 with the lengthened cytoplasmic tail (fBST2*). Currently, the detection of BST2 mostly relies on the fusion of the epitope tag peptide in the N-terminus or an interior region. In order to determine the possible influence of a peptide tag on fBST2, an HA tag was either added to the N-terminus of the protein or internally in the ectodomain proximal to the GPI anchor as evaluated in a previous report [28] (Fig 1B).

### Preparation of effective pAb against fBST2

**Fig 2:**
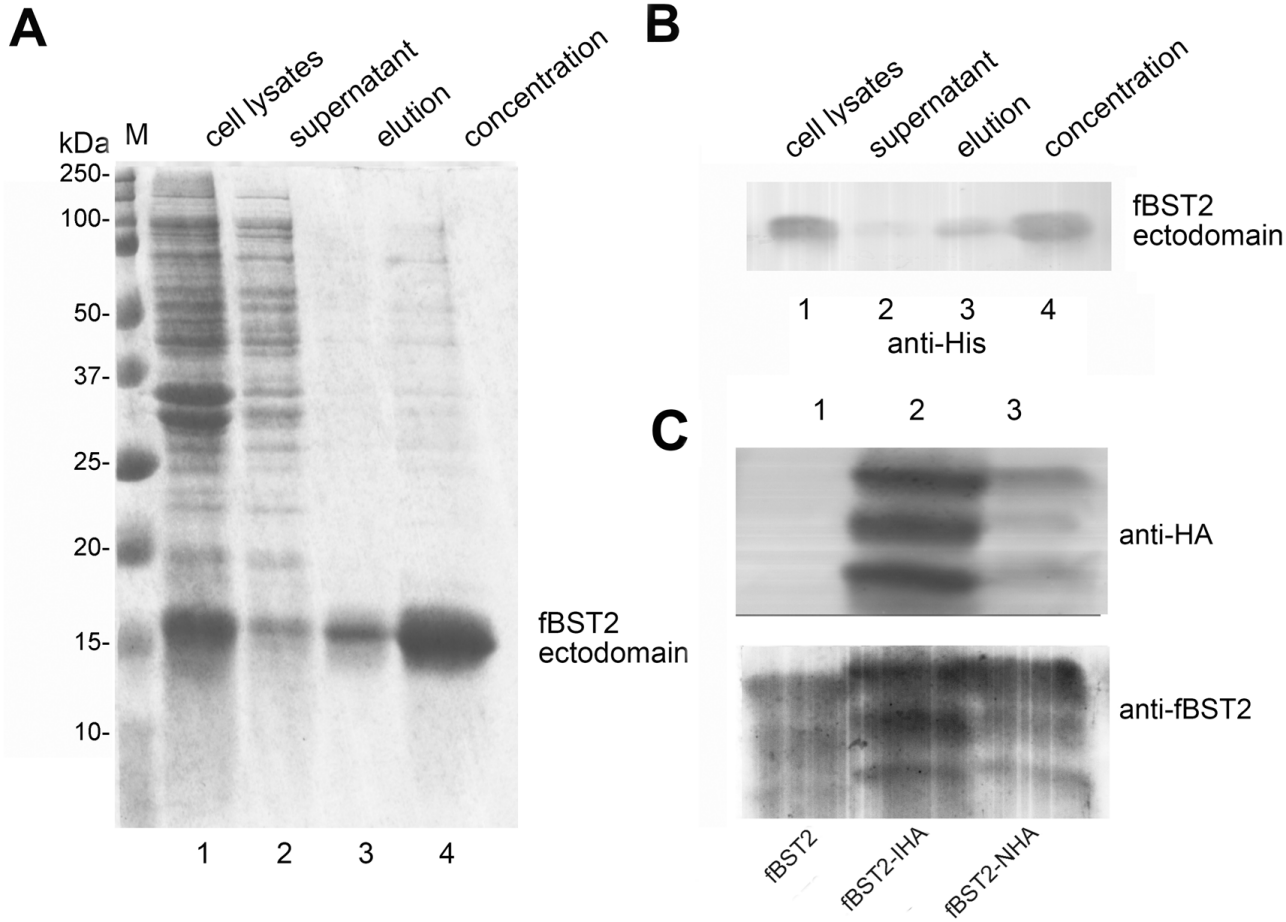
Purification of BST2 ectodomain and validation of antibody. (A and B) Purification of fBST2 ectodomain (15 kDa). The protein was purified by affinity chromatography from 6 L of *E*. *coli* BL21 transformed with fBST2 ectodomain expression plasmids based on the pET-26b (+) vector. Samples collected from the purification procedures were analyzed by SDS-PAGE with Coomassie Brilliant Blue staining (A) and Western blot (B) using an anti-His antibody. (C) Validation of pAb against fBST2. 293T cells were transfected with 500 ng of fBST2, fBST2-IHA and fBST2-NHA. After 48 h, cells were analyzed by Western blotting using the anti-fBST2 pAb and anti-HA mAb.

As no commercial products are available that can recognize untagged fBST2 and fBST2*, we prepared a pAb against fBST2. First, the prokaryotic expression plasmid of fBST2 was constructed. The cDNA encoding the fBST2 ectodomain, in which the N-terminus including the TM domain and C-terminal GPI anchor were both deleted leading to total elimination of the interaction between BST2 and the lipid membrane, was generated and inserted into the pET-26b (+) vector (Fig 1C). The resulting fBST2 ectodomain pET-26b (+) vector was transformed into *E*. *coli* BL21, and the expressed proteins were purified with nickel-chelating beads as described in Materials and Methods. Results of Coomassie Brilliant Blue staining and Western blotting indicated that the fBST2 ectodomain proteins in whole cell lysates were partially solubilized in the buffer (Fig 2A and 2B, lanes 1, 2). The expressed fBST2 ectodomain protein was about 15 kDa (Fig 2A). After elution and concentration, the purified protein was used for immunizing rabbits as described previously.

To validate the effectiveness of the anti-fBST2 pAb in the rabbit anti-serum obtained above, 293T cells were transfected with the fBST2, fBST2-IHA or fBST2-NHA plasmid. Forty-eight hours later, lysates of the 293T cells were separated by SDS-PAGE and analyzed by Western blotting using the anti-HA mAb and rabbit anti-serum against fBST2 as primary antibodies. The results indicated that the rabbit anti-serum and anti-HA mAb were equally capable of recognizing the fBST2 proteins, and each fBST2 protein showed remarkably different patterns (Fig 2C), potentially due to impairment of glycosylation. Therefore, we used the rabbit anti-serum as the anti-fBST2 pAb in subsequent experiments.

### Glycosylation patterns of fBST2 mutants differ from wild-type fBST2

Human BST2 migrates as a number of different sized species from around 28 to 36 kDa in SDS-PAGE analysis, presumably due to the heterogeneity in post-translational modifications, especially N-linked glycosylation. A previous study showed that mutation at both of the two putative N-linked glycosylation sites (N65A and N92A) reduced the apparent molecular weight of both transiently and stably expressed human BST2 to 21 kDa. Meanwhile, treatment of the stably expressed 32–38 kDa human BST2 protein with PNGase F reduced its molecular weight to a similar degree as that by mutation of both glycosylation sites [28]. Furthermore, the N79A/N119A mutant of fBST2 without glycosylation was previously reported to almost completely lose its anti-viral activity [53]. These observations all suggest that N-linked glycosylation is very important for the anti-viral activity of BST2 proteins.

**Fig 3:**
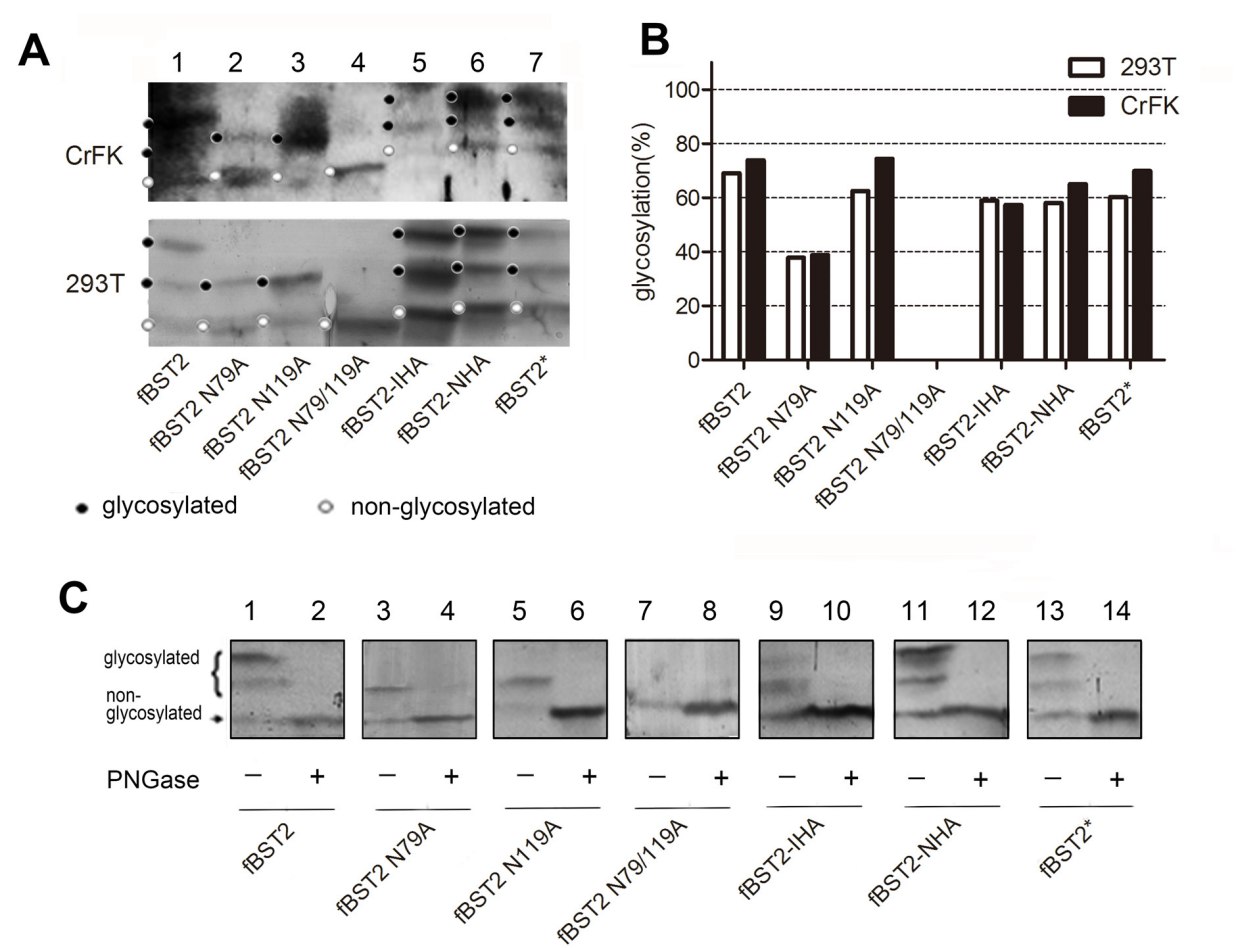
Detection of BST2 proteins and glycosylation analysis. (A) SDS-PAGE analysis of BST2 mutants under denaturing conditions. 293T cells and CrFk cells were transfected with 500 ng of fBST2, fBST2 N79A, fBST2 N119A, fBST2 N79/119A, fBST2-IHA, fBST2-NHA and fBST2*. After 48 h, cells were harvested and analyzed by Western blotting using the anti-BST2 pAb. The major bands corresponding to higher and lower glycosylated BST2 are marked with symbols. (B) Blots of BST2 in A were quantified using Bandscan software. The higher and lower glycosylated BST2 bands are shown in a column diagram, and all three bands of each BST2 were treated as 100%. (C) The fBST2, fBST2 N79A, fBST2 N119A, fBST2 N79/119A, fBST2-IHA, fBST2-NHA and fBST2* plasmids (each 500 ng) were transfected into 293T cells. Cells were harvested 48 h after transfection and divided into two portions. Cell lysates were incubated in the presence or absence of PNGase F and analyzed by Western blotting using the anti-fBST2 pAb. This experiment was repeated three times, and the most representative data are shown.

**Fig 4:**
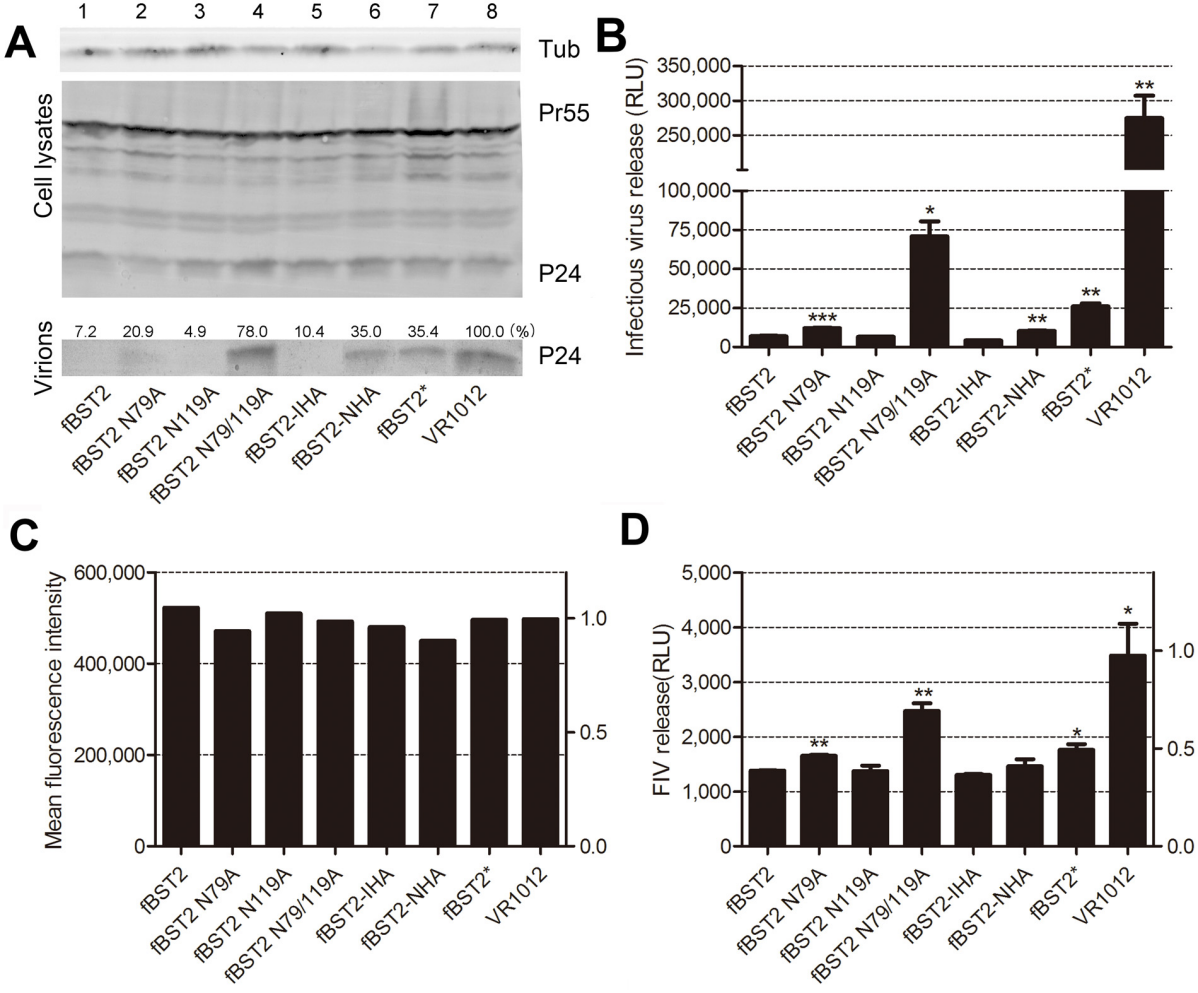
Effect of BST2 variants on HIV-1 virus and FIV pseudovirus particle release. (A) Twenty-five nanograms of fBST2, fBST2 N79A, fBST2 N119A, fBST2 N79/119A, fBST2-IHA, fBST2-NHA, fBST2* or the VR1012 control vector was co-transfected with 1 μg of the proviral plasmid pNL4-3 WT in 293T cells. At 48 h post-transfection, cultured supernatants were ultracentrifuged to concentrate the virus particles. Virions and cell lysates were analyzed by Western blotting using an anti-p24 antibody to detect viral p24 and intracellular Pr55Gag proteins, as well as an antibody against tubulin to assess sample loading. The blots of released p24CA were quantified using Bandscan software and normalized by tubulin levels. (B) Titration of the released infectious viruses in A is shown in columns, which are representative of three independent experiments and expressed as relative luciferase units (RLU). (C) VR1012, fBST2, fBST2 N79A, fBST2 N119A, fBST2 N79/119A, fBST2-IHA, fBST2-NHA or fBST2* (25 ng each) was co-transfected with 1 μg of FIV-ΔVif-ΔOrfA-ΔEnv-GFP reporter virus plasmid (pFP93: pGINSIN: pVSV-G = 3:3:1) in CrFK cells. Forty-eight hours later, the cells were harvested and analyzed for fluorescence intensity by flow cytometry as an expression control. (D) Supernatants from producer cells were harvested, clarified and assayed for GFP fluorescence intensity using a fluorescence microplate reader to represent released virus yield. This experiment was repeated three times. *P < 0.05; **P < 0.01; ***P < 0.001 compared to fBST2.

In order to determine the importance of N-glycosylation of fBST2, we constructed the following N-glycosylation site mutants: fBST2 N79A, fBST2 N119A and fBST2 N79/119A (Fig 1D). When we analyzed the seven fBST2s transfected in 293T and CrFK cells by Western blot using the anti-fBST2 pAb, fBST2-IHA, fBST2-NHA and fBST2* showed remarkably different patterns from those of fBST2, fBST2 N79A and fBST2 N119A, which only had one N-glycosylated pattern, while fBST2 N79/119A showed no N-glycosylated patterns (Fig 3A). After transfection of 293T cells, cell lysates expressing fBST2 or each variant were treated with PNGase. Each PNGase-treated protein showed as a band of reduced molecular weight, which was decreased to the same position of the lowest band of the untreated sample (Fig 3C). These results suggested that the upper, middle and lower bands of fBST2 corresponded to multiple, single and nonglycosylated forms, respectively. The wild-type fBST2 was strongly glycosylated (~70%) (Fig 3A and 3B, lane 1; Fig 3C, lane 1), while fBST2 N119A, fBST2-IHA, fBST2-NHA and fBST2* were ~60% glycosylated (Fig 3A and 3B, lanes 3, 5, 6 and 7; Fig 3C, lanes 5, 9, 11 and 13). By contrast, fBST2 N79A showed a lower glycosylation level (~40%) compared with that of fBST2 (Fig 3A and 3B, lane 2; Fig 4C, lane 3), and fBST2 N79/119A was not glycosylated (Fig 3A and 3B, lane 4 and Fig 3C, lane 7). The conclusions were obvious when the bands were quantified as shown in the column diagram (Fig 3B). These results suggest that adding a peptide to fBST2 may impair its normal glycosylation process, even though it still contains all putative N-glycosylated sites.

### Anti-viral function of fBST2 is affected by peptide modifications

Recent studies indicated that fBST2, with a very short N-terminal cytoplasmic region compared with those of other mammalian and non-mammalian homologs, could block budding of FIV and HIV-1 [52, 53]. Meanwhile, fBST2* has exhibited only limited anti-viral activity [52, 54]. In this study, the HA-tagged fBST2 and N-glycosylation sites mutants were also analyzed to determine if they still retained anti-viral function. First, 293T cells were co-transfected with fBST2, different variants or VR1012 (empty vector) and pNL4-3. The cell lysates and released capsid proteins were then analyzed by Western blotting, and Pr55 Gag was detected as a transfection efficiency control (Fig 4A). The HIV-1 viral particles exhibited a significant decrease (~90%) of release from cells expressing fBST2 (Fig 4A, lanes 1 and 8) and fBST2-IHA (Fig 4A, lane 5). Conversely, cells that expressed fBST2-NHA showed an impairment of ~35% in capsid protein release (Fig 4A, lane 6). By contrast, the extended fBST2 cytoplasmic tail truncation mutant fBST2* displayed an attenuation (~60% decrease) in the efficiency of anti-viral particle release compared with that of fBST2 (Fig 4A, lane 7). Compared to the wild-type, N-glycosylation sites mutated fBST2 N79/119A only had ~20% anti-viral activity (Fig 4A, lane 4); meanwhile, fBST2 N79A maintained ~20% of the ability to inhibit virus release (Fig 4A, lane 2), and fBST2 N119A has a strong anti-viral activity (~90%) (Fig 4A, lane 3).

Next, different fBST2 plasmids or VR1012 were co-transfected with the FIV-ΔVif-ΔOrfA-ΔEnv-GFP virus plasmid (pFP93, pGINSIN and pVSV-G) into CrFK cells. The cells and supernatants were analyzed as an expression control (Fig 4C) and to evaluate virus release (Fig 4D). Similar to the decrease in HIV-1 release, fBST2 effectively decreased FIV release (~40%) (Fig 4D, lanes 1 and 8). The two HA-tagged fBST2 mutants, fBST2-IHA and fBST2-NHA, showed little change (~40% and ~45% decrease, respectively) in the efficiency of viral particle release compared with that of fBST2 (Fig 4D, lanes 5 and 6), while fBST2* still maintained ~50% of the capability to inhibit virus release (Fig 4D, lane 7). Among the three N-glycosylation site mutants, fBST2 N119A could still inhibit virus release well (~40%), while fBST2 N79A and fBST2 N79/119A lost much anti-viral activity against FIV pseudovirus (Fig 4D, lanes 2, 3 and 4). The above results indicated that the N-terminal HA peptide and fBST2* peptide had a negative impact on the anti-viral function of fBST2; meanwhile, fBST2 with an internal HA tag maintained a good level of anti-viral activity, and the N-glycosylation mutants showed different levels of anti-viral activities. Furthermore, differences in degrees of glycosylation had no significant effect on the anti-viral activity of fBST2 as long as the glycosylation residues remained.

### Similar cellular locations of fBST2 variants

**Fig 5:**
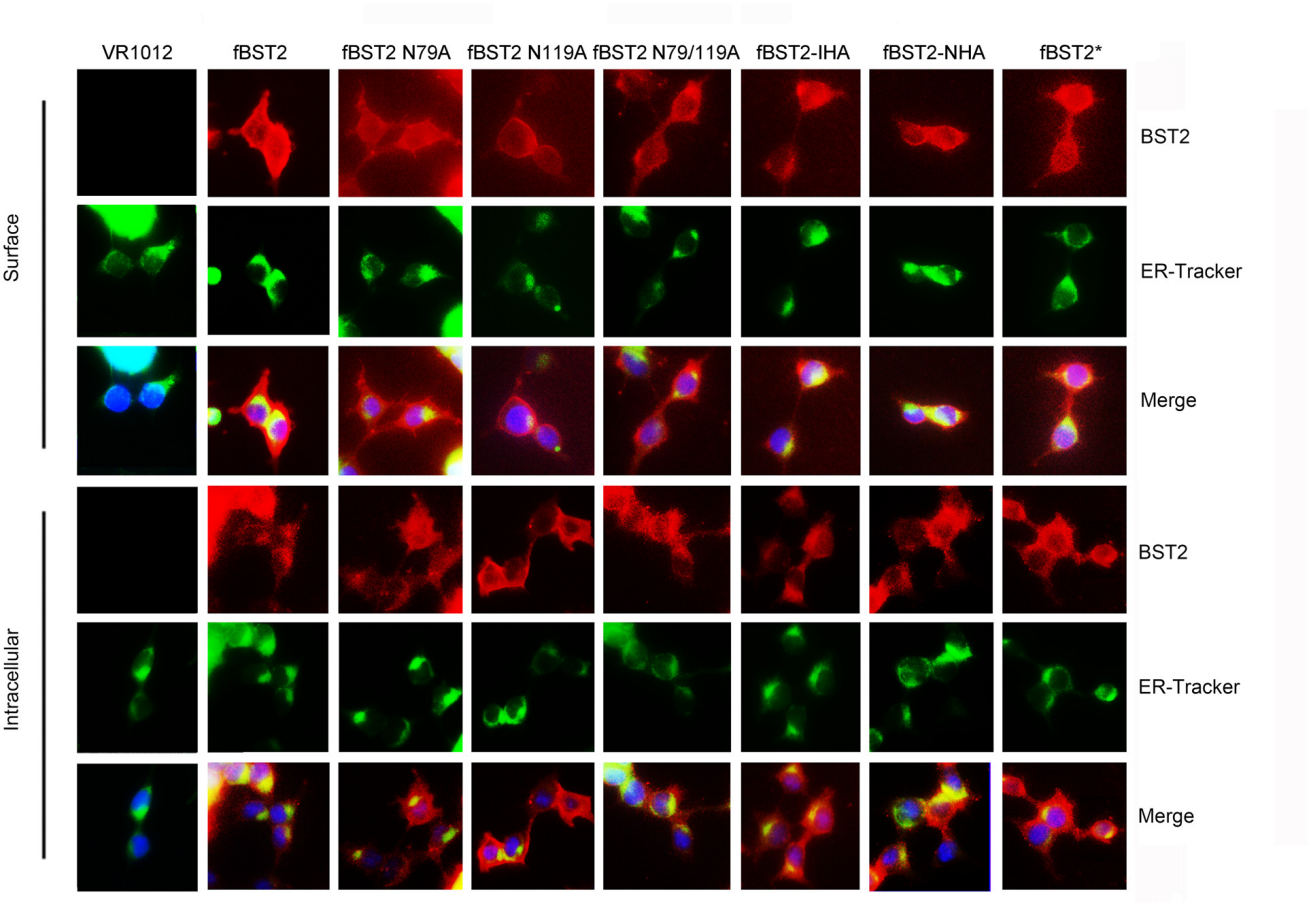
Intracellular localization of BST2 variants. 293T cells transfected with 200 ng of control plasmid VR1012 and expression plasmids fBST2, fBST2 N79A, fBST2 N119A, fBST2 N79/119A, fBST2-IHA, fBST2-NHA and fBST2* were observed by confocal microscopy with surface staining and intracellular staining: blue, cell nucleus; red, BST2 protein; green, ER. At least 30 independent cells were examined in each sample, and the most representative cells are shown.

BST2 is constitutively recycled between the plasma membrane and the endosomal and trans-Golgi network (TGN) compartments [59], although it has been shown to localize mainly to the plasma membrane and exhibit a puncta-like distribution [24]. In the current study, subcellular distributions of fBST2 mutants were detected to verify if the alteration of glycosylation was associated with changes in cellular localization. Subconfluent monolayers of 293T cells were transfected with fBST2, its variants or VR1012. Surface and intracellular protein localization patterns were evaluated by an indirect confocal immunofluorescence assay using the anti-fBST2 pAb. As shown in Fig 5, surface distribution levels of fBST2 N79A, fBST2 N119A, fBST2 N79/119A, fBST2-IHA and fBST2-NHA, as well as the wild-type fBST2, were obvious and considerable. Meanwhile, their intracellular patterns exhibited no differences, and all the proteins could co-localize with the endoplasmic reticulum (ER). These results showed that the intracellular and cellular protein localizations of the seven proteins were similar and not associated with alterations of glycosylation levels, nor were they related to anti-viral activities.

### Reduction in cell surface expression of N-terminally modified fBST2

**Fig 6:**
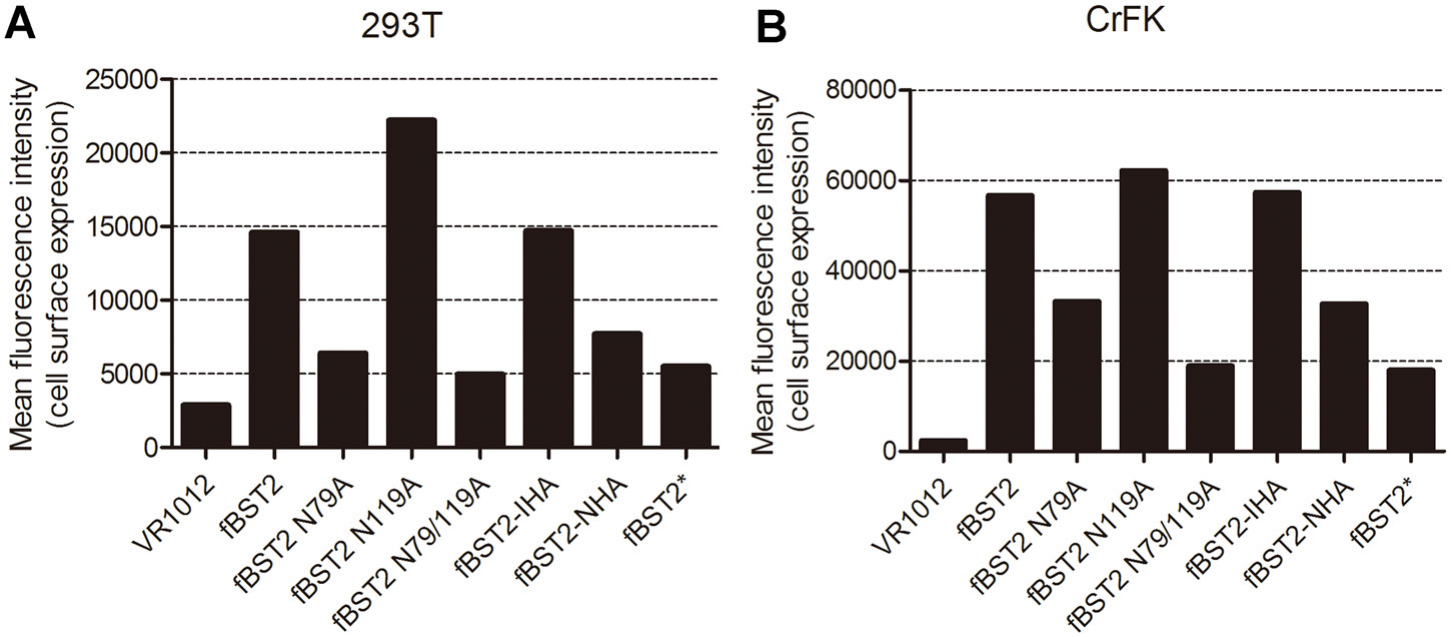
Cell surface expression of BST2 variants. 293T and CrFK cells were co-transfected with 500 ng of VR1012, fBST2, fBST2 N79A, fBST2 N119A, fBST2 N79/119A, fBST2-IHA, fBST2-NHA or fBST2* expression plasmid, along with 500 ng of pEGFP-N3 as a transfection marker. After 48 h, cells were harvested and stained with the anti-fBST2 pAb and Alexa 633 goat anti-rabbit IgG, followed by flow cytometric analysis. Cells only transfected with pEGFP-N3 were used as a negative control. The samples were gated on EGFP^+^ cells, and the surface BST2 levels are shown in the column diagram with mean fluorescent intensity values (A and B). This experiment was repeated three times, and the most representative data are shown.

Most researchers accept that BST2 must appear at the cell surface to exert its anti-viral function. In order to investigate the effect on cell surface localization by adding a peptide to different regions of fBST2 or mutating its glycosylation sites, the following FACS analysis was carried out. The EGFP^+^ encoding vector pEGFP-N3, used to verify the transfection efficiency for FACS analysis, was co-transfected with BST2 or VR1012 in 293T and CrFK cells. By flow cytometric analysis, cell surface protein levels were evaluated with the anti-fBST2 pAb and Alexa-633 conjugated anti-rabbit secondary antibody. Cells transfected with pEGFP-N3 and VR1012 were used as negative controls. Samples were gated on EGFP^+^ cells, and the surface BST2 levels were compared in histograms (Fig 6A and 6B). The cell surface distribution of the N-terminally modified mutants fBST2-NHA and fBST2* decreased remarkably compared with fBST2, while internally HA-tagged mutants fBST2-IHA was similar to fBST2. Surface levels of N-glycosylation site mutants were different, fBST2 N119A had a higher surface level than fBST2, while fBST2 N79A and fBST2 N79/119A only had a low level. Notably, fBST2, fBST2 N119A and fBST2-IHA showed better anti-viral activities than did the other three variants (Fig 4), suggesting that the cell surface appearance of fBST2 is a condition of its anti-viral function.

### Wild-type fBST2 does not mediate activation of NF-κB

**Fig 7:**
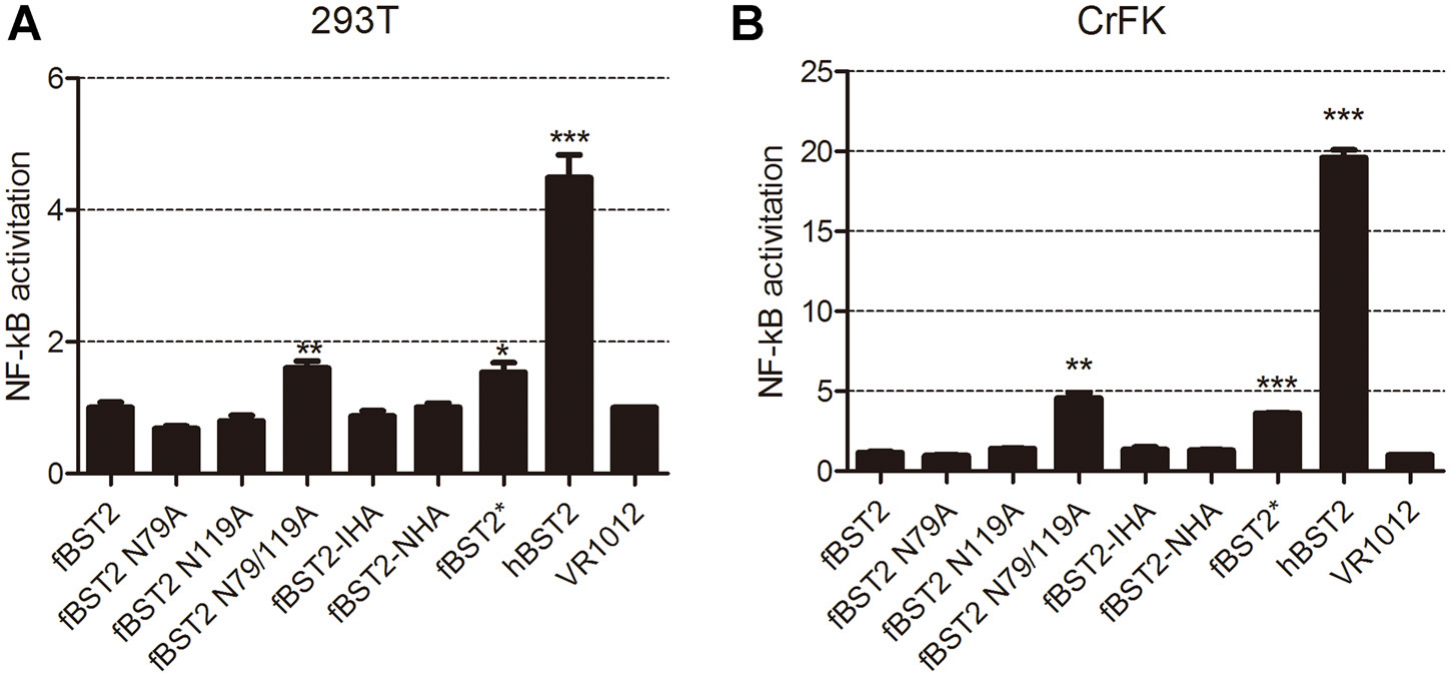
NF-κB activation mediated by BST2 variants. (A and B) 293T and CrFK cells were co-transfected with 25 ng of each variant BST2 expression plasmids separately, along with 250 ng of pNF-κB-Luc reporter plasmid and 50 ng Renilla luciferase plasmid. After 48 h, cells were lysed and analyzed for luciferase activity. The raw luciferase value was normalized to the Renilla luciferase value. Data are shown as fold changes in NF-κB activation compared with the mock control. The graph was generated from three independent experiments. *P < 0.05; **P < 0.01; *** *P* < 0.001 compared to VR1012.

Although primarily characterized as an intrinsic cellular viral restriction factor, BST2 has been proposed to have additional activities, including a role in induction of the pro-inflammatory response regulator NF-κB [25, 26, 27]. To explore if the wild-type fBST2 with a short N-terminus would have the ability to activate NF-κB, 293T and CrFK cells were co-transfected with the wild-type or mutated BST2 expression plasmid and luciferase reporter plasmid pNF-κB-luc along with a Renilla luciferase plasmid as a transfection control. The human BST2 plasmid was used as a positive control. At 48 h post-transfection, the cell lysates were collected and analyzed for luciferase activity. NF-κB activation with the human BST2 positive control was increased by approximately 5-fold in 293T cells and 20-fold in CrFK cells, compared with the mock control cells transfected with the expression vector alone, while it was increased by 1.5-fold in 293T cells and 4-fold in CrFK cells by fBST2*. By contrast, activities of fBST2, fBST2-IHA and fBST2-NHA were negligible (Fig 7). These observations suggest that the wild-type fBST2 was not able to mediate activation of NF-κB; however, the artificial fBST2* mutant containing a longer N-terminal cytoplasmic homolog with the YxY motif could activate NF-κB weakly. Although it does not have the YxY motif, fBST2 N79/119A could also activate NF-κB, while fBST2 N79A and fBST2 N119A could not. We surmised that since fBST2 N79/119A cannot be N-glycosylated, it would have to cause some inflammatory reaction to activate the NF-κB pathway.

## Discussion

The predominant mechanism for BST2-mediated inhibition of viral particle release is the direct tethering of virus to cells. In this process, the TM domain and GPI domain of BST2 are responsible for binding the cell membrane with the viral membrane [28]. However, the function of the N-terminal cytoplasmic tail region of this protein is not quite clear. Dietrich and coworkers recently characterized a putative feline homolog of human BST2 and assessed its effect on viral replication [54]. That study was based on a protein containing the above-mentioned 19 amino acids upstream of the first methionine and, most likely, generated by the positioning of an ATG in the place of the GAG codon during the cloning process [54]. In subsequent research, fBST2 was found indeed to have a short N-terminal region that is counteracted by the FIV envelope glycoprotein [52, 54]. Based on the current knowledge, we suspect that fBST2 with the shorter N-terminal cytoplasmic tail may lack some functions compared with human BST2.

In order to understand the contribution of the fBST2 cytoplasmic tail region, we constructed a series of BST2-derived proteins (fBST2*, fBST2, fBST2-IHA and fBST2-NHA). By Western blot analysis, we found that fBST2, fBST2-IHA and fBST2-NHA displayed remarkably different apparent forms, potentially due to different degrees of glycosylation. The N-glycosylation sites of fBST2 were also mutated in order to study the importance of N-glycosylation for its function,

Based on those observations, the underlying mechanism for the effect of a peptide on the functions of fBST2 needed to be investigated. The glycosylation analysis indicated that adding a peptide to fBST2 may impair its normal glycosylation process, although it still contained all putative N-glycosylated sites. This result implied that the original trafficking pathway of the native protein may undergo unpredictable changes, subsequently leading to the rearrangement of the intracellular distribution and alteration of surface expression, which can lead the decrease of the antiviral activity.

In virus release assays, the internally HA-tagged fBST2 protein showed an equivalent level of activity against HIV-1 compared with the wild-type fBST2. Meanwhile, the internally HA-tagged and untagged fBST2 could also inhibit the release of FIV-ΔVif-ΔOrfA-ΔEnv-GFP viral particles. Furthermore, addition of a tag to the N-terminus of fBST2 (fBST2-NHA and fBST2*) resulted in serious impairment of anti-viral activity with both HIV-1 and FIV-ΔVif-ΔOrfA-ΔEnv-GFP viral particles compared with the wild-type protein. When the N-glycosylation sites were mutated, fBST2 showed different anti-viral capacities. This phenomenon demonstrated that the cytoplasmic region of fBST2 may have an impact on its anti-viral function, as the fBST2 proteins with a modified cytoplasmic tail showed a weaker effect on HIV-1 and FIV-ΔVif-ΔOrfA-ΔEnv-GFP viral particle release, and the degree of N-glycosylation was not the vital component for anti-viral activity. These results were partially consistent with earlier findings emphasizing the importance of the cytoplasmic tail region to the anti-viral function of human BST2 [24, 52, 53]. Altogether, these findings suggest that the fBST2* peptide of fBST2 is functionally unnecessary, at least for its anti-release activity.

The immunofluorescence analysis showed that the cellular distribution of fBST2s were the same, and they could all localize to the ER. Meanwhile, flow cytometry analysis confirmed that the cell surface distribution of the N-terminally tagged mutants fBST2-NHA and fBST2* decreased remarkably; meanwhile, the internally HA-tagged mutant fBST2-IHA was similar to fBST2, and fBST2 N79A, fBST2 N119A and fBST2 N79/AA9A differed greatly in surface expression. We speculated that the additional sequence in the N-terminus retained the misfolded protein in the ER primarily and prevented transit through the secretory pathway. Mutation of N-glycosylation sites also could influence the post-translational modifications, leading to the retention in ER. These abnormal processes finally caused the insufficient protein trafficking and reduction of cell surface expression, which may contribute to the decline of anti-viral activity.

Human BST2 has been proposed to play a role in induction of the pro-inflammatory response regulator NF-κB [25,26,27]. The luciferase activity analysis showed that the wild-type fBST2 had an impaired ability to activate NF-κB. The determinants of NF-κB activation are currently defined as a tyrosine-based motif (YxY) and a RVP (10–12) motif found within the cytoplasmic tail of human BST2 [46–48], which should explain the loss of function for fBST2, fBST2-IHA and fBST2-NHA. Moreover, fBST2* and fBST2 N79/119A led to approximately a 1.5-fold or 4-fold increase in the NF-κB activation level over mock control cells transfected with the expression vector alone, although the activities were much weaker than that of wild-type human BST2. These results confirmed that the function of NF-κB activation is distinct from the restriction of virus release.

In conclusion, our results provide evidence that although the fBST2 has a much shorter N-terminal cytoplasmic region compared with those of other mammalian and non-mammalian homologs, it still retained anti-viral activity against HIV-1 and FIV pseudovirus viral particle release. However, the lost amino acid sequence was demonstrated to have the ability to mediate activation of NF-κB. Furthermore, fusion of a peptide tag to different positions on the fBST2 protein could impair its anti-viral activity to varying degrees. The HA tag and fBST2* peptide in the N-terminal cytoplasmic peptide may have affected the association of the recombinant BST2 with lipid membranes such as ER retention. Such impairment in intracellular trafficking reduced localization of the N-terminally tagged fBST2 to the cell surface, eventually weakening its anti-viral function. Meanwhile, mutating both N-glycosylation sites of fBST2 greatly reduced its anti-viral activity, but the influence of the single site mutant was slight. Importantly, these observations demonstrate that the fBST2* peptide was abandoned through the process of evolution, as it had a minimal impact on wild-type fBST2 and even maintained an ability to activate NF-κB. While the anti-viral function, N-glycosylation and ability to activate NF-κB of fBST2 may be three independent biological functions, its anti-viral activity is related to its cell surface expression. These results provide additional details and implications for the structure-function model of BST2. Additionally, these findings highlight the importance of the choice of tag position in functional studies of biological molecules.
